# Engaging patients in patient safety: a qualitative study examining healthcare managers and providers’ perspectives

**DOI:** 10.1186/s12912-022-01152-1

**Published:** 2022-12-29

**Authors:** Samaneh Sarkhosh, Zhaleh Abdi, Hamid Ravaghi

**Affiliations:** 1 Master of Health Services Administration, School of Health Management and Information Sciences, Iran University of Medical Sciences (IUMS), Tehran, Iran; 2 National Institute of Health Research (NIHR), Tehran University of Medical Sciences, Tehran (TUMS), Tehran, Iran; 3 School of Health Management and Information Sciences, Iran University of Medical Sciences (IUMS), No. 6, Rashid Yasemi St. Vali-E-Asr Ave, P.O Box: 1996713883, Tehran, Iran

**Keywords:** Patient engagement, Patient safety, Qualitative study, Hospital, Iran

## Abstract

**Background:**

Patients can play an essential role in improving patient safety by becoming actively involved in their health care. The present study aimed to qualitatively explore healthcare providers’ (HCPs) and managers’ perceptions on patient participation in patient safety processes.

**Methods:**

This qualitative study carried out in three teaching hospitals in Tehran, Iran. The data were collected through semi-structured interviews with 31 HCPs and managers working at public teaching hospitals, medical universities and the Ministry of Health. The data were analyzed using thematic analysis.

**Results:**

Three main themes and 21 sub-themes emerged from the interviews. Participants believed that patients and their families can play an effective role in maintaining and improving patient safety through different roles. However, a variety of barriers were identified at patients, providers, and system levels hindering patient participation in delivering safe care.

**Conclusion:**

The participants identified facilitators and barriers to patient engagement in safety-orientated activities at multiple patients, providers, and system levels, indicating that complex, multifaceted initiatives must be designed to address the issue. This study encourages further research to enhance the understating of the problems and solutions to patient involvement in safety initiatives in the Iranian healthcare setting.

**Supplementary Information:**

The online version contains supplementary material available at 10.1186/s12912-022-01152-1.

## Introduction

Patient safety is a significant public health issue [1]. It has been globally estimated that approximately one in 20 patients are harmed while receiving medical care in primary, secondary, and tertiary care settings worldwide, while half of these harms are preventable [2]. Traditionally, strategies to improve safety have focused on solutions involving the actions of institutions or professionals, including developing incident reporting systems, changing systems of care, and professional behavior [3]. However, there has recently been a growing interest in involving patients and their representatives in safety initiatives [4–6]. Internationally, several safety initiatives have highlighted the importance of patient engagement in patient safety [7–9]. In 2004, the World Health Organization (WHO) identified patients, families and community engagement as one of six initial patient safety priorities, and it continues to be a core priority of WHO patient safety initiative [10]. It has been widely recognized that an effective approach to understanding and preventing medical errors must account for the roles and actions of all actors involved in system processes [11]. Recent evidence shows that patients could make essential contributions to their safety and prevent errors and adverse events. They can play a significant role in reaching an accurate diagnosis, deciding appropriate treatment, choosing the providers, making sure that treatment is administered as planned, and detecting incidents and acting to prevent them [12]. Patient engagement in patient safety seeks to increase the awareness and participation of patients in error-prevention strategies [3, 13]. However, many factors hinder patient participation, including acceptance of the new role of the patients by caregivers, unwillingness of patients, cultural barriers, and lack of system-level efforts supporting patient and family engagement [14]. Thus, there is clearly a need to understand barriers and challenges of patient involvement in patient safety.

In recent years, a range of interventions, including Clinical Governance initiative in hospitals [15, 16], the nationwide introduction of clinical risk management and root cause analysis [17], national hospital accreditation system, and the WHO Patient Safety Friendly Hospital Initiative (PSFHI) [18] have been planned and executed in Iran to improve quality and safety of healthcare services provided in hospitals. The introduction of clinical governance initiative (2009) has increased the emphasis placed on patient safety through structural and procedural changes at the hospital level in Iran [15]. The program requires hospitals to plan for and establish systems to minimize risks proactively and investigate causes of sentinel or adverse events using root-cause analysis [17]. More specific, the program trained managers and clinical staff at the university and hospital levels in leadership of patient safety and quality improvement to improve safety culture [16]. In addition, Ministry of Health and Medical Education (MoHME) issued supporting regulations and guidelines such as hand hygiene, safe surgery checklists and patient safety walk-round guidelines in order to facilitate the hospitals’ continuous attention to patient safety. Besides, patient safety indicators and standards were included in the national hospital accreditation program, which required hospitals to incorporate the standards into their daily work schedules [19]. Further, specific research and improvement projects were developed to identify and address relevant challenges in hospital settings across the country, which led to a growing body of research on patient safety. However, despite this growth and despite the international emphasis on patient involvement in safety activities, evidence is lacking on clinicians’ and patients’ perspectives toward active engagement of patients in safety. To shed light on this issue, the present study aimed to qualitatively explore healthcare providers (HCPs) and managers’ views and perceptions towards patient participation in patient safety processes, and to investigate perceived barriers and facilitators to the participation in practice.

## Methods

### Study design

A qualitative approach with a semi-structured interview guideline [20] was applied to provide insights into the attitudes and perceptions of frontline HCPs and managers working at hospitals, medical universities and the MoHME regarding patient engagement in patient safety.

### Data collection

A topic guide was developed to collect the data based on the existing literature and experts' opinion (Additional file 1). There were general questions on respondent understanding of patient safety. There were also specific questions on the respondents' attitudes about the idea that patients/families could have a role to play in enhancing their safety while staying in hospital. The interview guide ended with questions on barriers and facilitators to patients’ involvement in patient safety initiatives from the respondents’ perspective. The topic guide was piloted through two interviews, and some amendments were made. The pilot interviews feedback was used to finalize question wording and determine question order. The test interviews were not included in the study. Data was collected through individual semi-structured interviews (from September to November 2019) conducted by the first author at the participants’ workplaces. Each interview took 45–60 min on average. Each interview was digitally recorded and transcribed verbatim by a professional transcriber into English.

### Participants

The interviewees were selected from three teaching hospitals affiliated with public medical universities in Tehran, Iran. A purposive sampling strategy [21] was used to include a mix of different healthcare professional disciplines, levels of experience, and position/role within the organization. The aim was to achieve a sample of HCPs that represented a broad spectrum of perceptions and experiences concerning patient involvement in relation to patient safety. To further understand the barriers and challenges of involving patients in patient safety initiatives, patient safety managers working at medical universities and the MoHME were approached. The interviews continued until data saturation was reached, with the final few consecutive interviews generate no new codes [22]. In total, 31 individuals were interviewed, including 14 frontline HCPs (7 physicians and 7 nurses), 4 hospital patient safety officers, 3 hospital quality improvement managers, 3 matrons, 3 hospital directors and 4 patient safety managers working at the medical universities and MoHME.

### Data analysis

Data was analysed manually following six phases of thematic analysis as described by Braun and Clarke [23]. The process included (1) familiarisation of data whereby transcripts were read and reread, (2) generating initial codes, (3) searching for themes by gathering relevant codes, (4) reviewing themes, (5) defining and naming themes, and (6) writing the report. The transcribed interviews were read several times by authors to obtain an understanding of the whole data. The first author coded data, which was re-checked and discussed by two other authors to ensure consensus on coding practices. The first author generated initial themes from the data, then consulted with two other authors for feedback and consensus on the naming and grouping of sub-themes. The authors then compared and discussed them in order to reach agreement. For member checking, a summary of the themes and subthemes, a copy of each participants’ interview summary, and a request for feedback were distributed among all participants. All 31 participants agreed with their transcripts and the researchers’ interpretation of emerging themes so only one round of member-checking was completed. To ensure research credibility, several steps were taken: prolonged engagement, data triangulation, and member checking. All authors were involved during the analysis stage, this allowed for a more thorough and well-rounded analysis of the data. Data triangulation was performed by reviewing relevant policy documents, and member checking was performed with participants' validation.

### Ethical considerations

Ethics approval was obtained from the Ethics Committee of Iran University of Medical Sciences (IUMS/SHMIS-1395/9311564011). All of the participants were informed of the objective of the study and the right to withdraw from the study at any time during data collection. Informed consent was obtained from those who agreed to participate in person. Further, participants were reassured that their responses would be kept confidential and their identities would not be disclosed in any resulting publication.

## Results

Three main themes and 21 sub-themes emerged from the interviews, including the role of patients and families in patient safety, and barriers to and facilitators of patient participation (Table 1).

**Table 1:** Identified main themes and subthemes from the semi-structured interviews

Theme	Subtheme/category	
Role of patients, families, and caregivers in patient safety	Role of patients	Active involvement in care
		Self-care and being compliant
		Speaking up and reporting errors
	Role of families and caregivers	Providing information about the patient to the medical team
		Speaking up and reporting errors
		Delivering quality at-home care
Barriers to patient engagement	Barriers related to patients	Lack of patients’ awareness regarding their role
		Low health literacy and lack of knowledge
		Lack of patient trust in health system and providers
		Cultural beliefs
		Socioeconomic backgrounds
		Linguistic and communication barriers
	Barriers related to healthcare providers	Unwillingness to involve patients
		Time constraints and high workload
	Barriers related to the health system	Healthcare setting -related barriers
		Educational barriers
		Lack of resources barriers
		Organizational cultural barriers
		Communication barriers
Facilitators of patient engagement	Increased awareness and knowledge of patient and healthcare providers	
	Promoting patient participation in patient safety as an international priority	

## Role of patients, families, and caregivers in patient safety

Participants considered the provision of safe care as a major goal of healthcare organizations. They believed that all healthcare workers, including clinical professionals and non-clinical staff, have a shared responsibility to promote patient safety. They asserted that although patients and families can play an important role in ensuring patient safety during their care, the responsibility for their safety must remain with the healthcare workers and organizations.

### Role of patients in patient safety

Three different roles were perceived for patients in improving patient safety: “active involvement in care,” “self-care,” and “speaking up and reporting errors”. Participants also discussed the value of patient involvement in patient safety.

Most participants (28 out of 31) noted that patients should be actively involved in all stages of hospital care as they are in the central position of the healthcare process. Participants mentioned some benefits for patient engagement, including better outcomes, greater levels of safety, improved satisfaction, fewer complaints, and reduced harms. By being involved in the delivery of their care, patients could also be involved in the safety of their care. This includes different activities in the clinical context, such as providing their medical histories precisely, attending follow-up appointments, taking and managing medications, and engaging in discharge planning. Nurses shared examples of situations when the involvement of the patients led to improvements in patient safety. The examples included patients reminding about allergies, asking for aids to avoid fall injuries, observing defects in medical devices, and asking about referrals that their providers had forgotten about. Participants saw these roles as examples of patients taking ownership over their health and safety or ways of involving patients in their safety.

Being compliant with the advice they received from HCPs and taking care of themselves were raised as another role of patients in improving patient safety. Participants asserted that when patients are concerned about their care, they comply with the instructions or advice about their care and safety processes within hospitals. Failure to comply with these instructions and advice can compromise the safety of the patients.

*“For example, non-compliance with prescribed treatment or medicine is an important cause of adverse events. I have seen patients stop their medication on their own without our permission.” (P 12, Physician)*

Another role perceived by participants was speaking up and reporting errors through asking questions about their care, checking different aspects of their care, and noticing and reporting errors. Participants considered that well-informed patients could observe and notice safety concerns and may draw HCPs attentions to things overlooked by HCPs during care provision. They can be regarded as a second layer of safeguards for medical errors and may even catch potential mistakes from their providers.

*"If the patients themselves recognize near misses, they can report the cases…. they can report that they were about to be given the wrong medicine. There were times that the staff did not notice the error or not report it, but the patient reported.” (P 19, Hospital matron)*

Mangers at the university and ministry levels stressed the importance of the increased interactions between patients and patient organizations, professional associations, and educational institutions. They believed that patients could share their experiences on adverse events and safety issues through these networks, thereby increasing their awareness of patient safety.

A commonly shared opinion was that although patient involvement in safe care is important, it is not well established in Iran’s health system. Some participants (11 out of 31) argued that there were no systematic interventions to raise the role of patients in patient safety processes. They believed that patient involvement in delivery of safe care would not occur unless healthcare organizations communicate these roles to their patients at the system level.

*“To achieve patient involvement, hospital leaders need to shift the institutional culture that has historically limited patient engagement*.” *(P 23, Physician)*

### Role of family and caregivers in patient safety

Participants pointed out that patients and their families have roles in achieving patient safety. They believed that the role of patients’ caregivers is very effective in cases where the patients are vulnerable (i.e., due to age or disability), confused, or unconscious. They considered patients without family and carers at increased risk of medical errors.

*“Vulnerable groups definitely need to have their families involved. It depends on the patient’s age, consciousness, and awareness. For a child patient, a patient who has had a stroke or has mental health problems, family involvement is critical.” (P 20, Hospital manager)*

Participants described a range of particular roles for the patients’ families and caregivers to ensure patient safety, including providing information about the patient to the medical team, speaking up and error reporting, and delivering quality at-home care to prevent hospital readmissions. Participants expressed that family and caregivers have a role in patient safety by providing accurate information to HCPs about the patient, which is much more important in confused and unconscious patients. Another role considered for family and caregivers was speaking up and reporting medical errors. Participants believed that in addition to accompanying the patient during the hospital stay, caregivers play a crucial role in the discharge process and support the patients at home.

“*When patients return home, they may have post-operative infections. When they [relatives] were informed, they would wash their hands, or check the site of surgery and in case of any complications, take patient to the hospital.” (P 11, Patient safety officer).*

## Obstacles to patient engagement in patient safety

The participants identified three categories of barriers related to patients, HCPs, and health system for patient engagement in safety activities.

### Barriers related to patients

Most participants (22 out of 31) pointed out that patients are not entirely aware of their rights while staying at the hospital, including the right to be involved in care planning and treatment. Lack of patients’ awareness regarding their role in patient safety improvement was identified as a major barrier, which can reduce their contribution level. They believed that the level of patients’ awareness regarding the process of care, including the possibility of medical errors and the associated harms as well as the role of patients in the detection and elimination of medical errors should be increased. However, some participants asserted that the level of awareness varies among individuals, and HCPs should pay attention to these differences in patients’ awareness and ability to learn.

*“The level of patients’ awareness (on their role on patient safety) is relatively low, affecting their participation; therefore, it does not mean that they do not want to be involved, but they cannot participate in the process because of their low level of information." (P1, Quality improvement officer)*

A number of participants (12 out of 31) believed that boosting patient engagement in their own care and safety could be challenging in Iran, where most patients have an assumed trust in physicians and the safety of care and do not want to undermine it. Most patients view HCPs as authority figures and are not likely to question their approach. Some patients are reluctant to ask questions, seek clarification, or request that information be repeated for fear of wasting HCPs' time.

*“There are some cultural barriers rooted in beliefs about authority and power. For example, challenging those in charge, such as doctors, is regarded as disrespectful in Iran. Thus, some patients may avoid asking questions, especially older or more traditional patients.” (P 25, Physician).*

Some participants (9 out of 31) believed that patient involvement in healthcare might be influenced by social norms, which are not within the control of either the patient or the healthcare worker. They asserted a need for community-driven strategies to develop a culture of participation in health delivery and health policy-making among community members. Some participants believed that a low level of patient participation and involvement could be associated with their socioeconomic backgrounds, such as age, sex, and level of education. They noted that young and highly educated patients or those with high socioeconomic backgrounds are more likely to participate in their care plan.

A number of participants (11 out of 31) argued that referral hospitals in large cities provide services to patients from diverse racial, ethnic, and cultural backgrounds. They considered language barriers as another obstacle to effective communication between HCPs and patients that may lead to errors. Patients’ lack of proficiency in the national language was regarded as a considerable barrier to patient engagement by several participants.

“*In referral hospitals located in Tehran, many patients from all over the country with different languages and dialects come. Some of them can hardly speak Farsi…. Language can be a major barrier to communication.” (P11, Patient safety officer)*

### Barriers related to healthcare providers

Participants postulated that HCPs’ unwillingness to involve patients is a significant obstacle, which might be due to personal beliefs, legal issues, and time constraints. Some participants believed that HCPs have not fully realized the importance of patient engagement in safety activities and, in turn, have not accepted the new role for patients. They believed that patient engagement in care and treatment could lead to increased patient expectations and, consequently, increased workload.

Nurse participants believed that physicians are reluctant to involve patients in making treatment decisions and care because of fear of legal liability. According to them, most physicians perceive that patients cannot understand medical information and providing them with such complex information is either ineffective or would create confusion and concern. In addition, some participants (9 out of 31) assumed that the current level of physicians’ engagement in quality improvement and safety initiatives such as hospital accreditation program is relatively low.

Time constraint was brought up by a number of participants (10 out of 31) as a barrier to involving patients in their care and safety issues. They asserted that the involvement of patients requires communication about safety which in turn needs time. This lack of time can result in less detailed explanations, unanswered questions, and patients feeling ignored and reluctant to be more involved in the care process.

*“Sometimes I’d really like to stay a little longer with the patient and answer his/her questions, but I am thinking in the back of my mind that there are lots of things that have to be done right now….” (P 26, Nurse)*

### Barriers related to the health system

Within this theme, participants mentioned five types of barriers to patient engagement, including healthcare setting-related barriers, educational barriers, resource constraints, cultural barriers, and communication barriers.

#### Healthcare setting related- barriers

Participants pointed out that some barriers have been embedded in the social or administrative structures of the health system. They believed that despite recent efforts to promote the patient safety concept in the Iranian health system, it still presents a significant healthcare issue. Patient safety and quality improvement officers mainly mentioned that although there were several national policies and regulations to make hospitals safer in recent years, most standards and regulations are beyond the capacity of hospitals and health facilities. They believed that reforms are mainly focused on structural changes rather than cultural and behavioral changes.

Some participants (10 out of 31) highlighted that due to the importance of the accreditation program for hospitals and the link between the hospitals' accreditation status and payments by health insurance organizations, hospitals have merely focused on receiving their certificates through documentation activities rather than the appropriate implementation of the standards and safety and quality improvement measures. Therefore, the reported scores on patient safety measures do not reflect their actual performance.

Lack of motivation among HCPs was perceived as another important system-related factor influencing patient engagement. Some participants (7 out of 31), particularly hospital managers and patient safety officers, pointed out that if hospitals compensated HCPs for their roles in safety and quality initiatives, there  would be more impetus for engagement and ownership. They hypothesized that greater engagement in safety efforts should be garnered by offering financial and organizational incentives.

#### Educational barriers

One of the most cited obstacles (17 out of 31) was the lack of relevant courses on patient safety and quality improvement for undergraduate health professions, including medicine, nursing, pharmacy, and dentistry, in their educational curricula. Participants believed that the development of skills, behaviors and attitudes regarding patient safety is of utmost importance for promoting safety culture for the next generation of HCPs. They argued that more education on patient safety and partnering with patients should be embedded in academic education and continuing professional development (CPD) programs. A few number of participants (6 out of 31) pointed out that the current educational system reinforces the paternalistic attitudes toward patients, affecting HCPs' willingness to engage patients in safety initiatives. However, some HCPs raised the issue that they had little support or were not qualified to engage patients, which was regarded as a system failure.

*“There is a lack of knowledge of patient safety and terminology among staff. It seems the system has not fully realized the importance of patient safety.” (P 7, Patient safety officer)*

The majority of participants (25 out of 31) noted that although patient education is key for patient engagement, public and patient education provided by teaching hospitals is not sufficient and effective. They believed that patients are not fully informed of their own rights, including the right to be involved in their own care during the informed consent process. Further, they believed that patients do not receive adequate education and counseling during their stay or discharge time.

#### Lack of resources barriers

Almost all participants (28 out of 31) identified scarcity of human resources, especially nursing staff, as a barrier to patient involvement in patient safety. They believed that this shortage causes HCPs to have less time for patients and makes it more difficult for them to maintain their entire focus and attention on the patient.

Financial constraints and lack of facilities were two barriers mentioned by a few number of participants (6 out of 31). Some patient safety officers mentioned that hospitals are currently suffering from financial difficulties and struggle to provide essential supplies such as safety boxes, personal protection equipment (PPE), and hand-washing solutions. They pointed out that the shortage in patient safety-related materials led HCPs not to take safety issues seriously.

#### Organizational cultural barriers

Most participants (23 out of 31) noted that patient-centered care has not been well-established in Iran’s health system. Some participants believed that the current organizational culture within healthcare organizations and even at the health system level is not patient-centric enough to allow patient engagement for patient safety sufficiently.

“*There is a health care culture in which the patient is seen more as a recipient of services than as a participant in care.” (P 20, Hospital manager)*

Some participants (10 out of 31) also indicated that public trust in physicians has declined in recent years due to propaganda brought about by social media, which consequently can impede any efforts to engage patients and family members in their care. In addition, a few number of participants (4 out of 31) at the top management level urged the need to change from a provider-centered mindset to a patient-centered care system, which involves rethinking the current policies, structures, and processes to promote patient engagement. In addition, several participants asserted that creating a culture of patient safety helps foster openness and transparency and may strengthen the patient-provider relationship.

“*The image of doctors has been damaged in recent years (by social media). They have been introduced as people just try to maximize their profits rather than improve their patients’ health and wellbeing…. This lack of trust can definitely lead to decreased patient engagement.” (P 18, Hospital manger)*

#### Communication barriers

Almost all participants (27 out of 31) identified communication barrier as a major obstacle to patient involvement. Participants' communication barriers included a lack of predefined routes or mechanisms to involve patients in patient safety activities and a lack of guidance documents regarding the legal and ethical responsibilities for HCPs during the process of involvement*.*

*“It is unclear whether it is our (doctors’) responsibility or nurses’ responsibility to involve patients, and if so, how we should do it.” (P23, Physician)*

In addition, poor physician‐patient communication was frequently mentioned (22 out of 31) as a barrier to patient participation. Participants emphasized that the relationship and communication between patients and families with members of the healthcare team, particularly physicians, are important for patients’ willingness to engage in safety-related behaviors. A poor relationship with HCPs makes patients less motivated to engage with their safety. Some participants argued that as different HCPs are involved in routine care delivery, it may impede the exchange of information between patients and providers and among providers on the healthcare team. However, some participants, including hospital managers and matrons, pointed out that hierarchical structure of the medical education in teaching hospitals hinder an effective relationship between physicians and patients to maintain patient safety. Some of them believed that public teaching hospitals care is more provider and practice-centered rather than patient-centered.

*“In public teaching hospitals, patients are visited by interns, residents, and fellows. They do not even know the attending doctor’ name who is in charge of their care.… some of these issues are normal in teaching hospitals where their first mission is educating medical and paramedical students”. (P7, Patient safety officer).*

## Facilitators of patient engagement in patient safety

Although participants perceived numerous barriers to patient engagement, they proposed several enablers to increase engagement. Participants believed that patients are more aware of their rights as consumers than ever and more literate about their health conditions and available treatment options. Hence, they are more willing to take a more active role in exercising their most fundamental rights. Participants believed that meaningful and effective engagement begins with empowering patients and HCPs. Almost all participants (29 out of 31) reported that educating patients and family members on patient safety is a key strategy for empowering them. They believed that patients and relatives need to have adequate information to make informed decisions about patients’ health conditions. They asserted that education should be provided through a variety of channels. They also believed that the health system should use the capacity of mass media to raise public awareness regarding the process of care, including the possibility of medical errors and the associated adverse effects as well as the role of patients and families in the reduction and elimination of such errors. Another identified facilitator was delivering education and training interventions to HCPs in order to change their knowledge, skills, attitudes, and behaviors regarding patient safety and patient engagement in safety activities.

*“I think patient safety officers should develop educational content to increase patient and family engagement in patient safety activities, such as personal patient education, large patient-safety campaigns, brochures, patient-safety videos, and other resources to increase patient engagement.” (P25, Physician).*

Participants, particularly nurses, asserted that due to the initiatives implemented by MoHME in recent years, the culture of health organizations has moved considerably from a punitive culture to a more just culture that encourages reporting and analyzing medical errors. Most participants acknowledged that the implementation of the patient safety initiatives endorsed and supported by the WHO in hospitals during recent years has brought the importance of the matter to the attention of health policymakers. The increased awareness led to the inclusion of patent safety standards in the national hospital accreditation program.

*“Due to these efforts, patient safety was included in the hospital strategic plan, which was significant progress …”. (P4, Patient safety officer)*

## Discussion

This qualitative study explored the views and perceptions of HCPs and managers on the involvement of patients and families in patient safety and investigated perceived barriers and facilitators to the participation. Consistent with similar studies, the participants expressed positive attitudes to patient and relatives’ involvement and believed it could have a positive impact on patient safety [24–26]. According to participants, the role of families and caregivers is more effective in cases where patients are not able to engage in their own health care. In our study, several roles were recognized by participants for patients and caregivers, such as “active involvement in care”, “self-care,” “speaking up and reporting errors,” “providing information about the patient to the medical team”, and “providing quality home care after discharge”, which were  consistent with those identified by previous studies [13, 27, 28]. A variety of barriers were identified at patients, providers, and system levels hindering patient participation in delivering safe care, which will be discussed below in detail.

The identified hindering factors associated with patient involvement for safer care are largely consistent with those identified in research on patient involvement from the patients and HCPs perspectives [24, 26, 29–31]. Participants believed that patients’ motivation to be involved in safe care was constrained by some factors, including low health literacy, lack of knowledge about their safety and rights, linguistic and communication barriers, and unwillingness to challenge providers’ knowledge and authority. Although some patient-related obstacles to patient participation are not within the control of either the patient or the healthcare organization, others could be addressed through appropriate strategies [32, 33]. Patient empowerment is key for a successful patient involvement, especially in error prevention strategies [34]. Patients need to have adequate information to make informed decisions about their own health and the care they receive [35, 36]. Similar studies on factors influencing patients’ engagement in their care reported that their engagement depends on their level of health literacy [26, 37, 38]. Studies have shown that individuals with low health literacy are less likely to use preventive health services and adhere to treatment recommendations; hence, they are more likely to experience the delayed diagnosis of medical problems and increased hospitalization, have a poorer health status, and a greater risk of mortality [39, 40]. In addition, relevant studies reported that individuals with limited health literacy are more likely to experience adverse events such as medication errors [41, 42]. Therefore, strategies developed to strengthen patient engagement should focus on improving health literacy [26, 28]. In countries such as Iran where health literacy in the general population is relatively low [43], more efforts are needed at the system level to develop a comprehensive response to the health literacy challenge in public and among patients.

We found several barriers related to HCPs, including personal beliefs, legal issues, and time constraints, leading to their unwillingness to engage patients. Similar barriers associated with HCPs were reported in the literature, including the desire to maintain control, lack of time to educate patients and caregivers, HCP knowledge and beliefs, HCP professional specialty, ethnic origin, and insufficient training and education in patient involvement [32, 44]. Time pressure was recognized as a barrier to patient participation for safer care in our study, which is consistent with previous similar studies [24, 45, 46]. Many changes in clinical practice have led to increased demands on physicians and nurses to document various aspects of their work, which along with staff shortage and heavy workload, have often been identified as key reasons for reduced time with patients in Iran’s hospital setting [47, 48]. This lack of time can result in less detailed explanations to patients, unanswered questions, and patients feeling ignored. Several studies showed that patients' motivation to participate in their own treatment and care is reduced when patients have concerns about being ignored, not believed, or not taken seriously [45, 49].

It is widely recognized that HCPs’ beliefs, attitudes, and behaviors can significantly affect patient participation [50, 51]. One of the major obstacles reported by the participants was the refusal of HCPs to abandon their traditional role and delegate power to patients, even though they may not openly oppose the idea of patient involvement. This finding is consistent with that of other similar studies exploring patients and HCPs’ perspectives on patient involvement [26, 32, 52]. Historically, in many cultures, the relationship between the patient and HCPs, particularly physicians, follows a “paternalist” model in which the patient has been a passive spectator in their own healing process [29, 32, 33]. In western countries’ healthcare systems, a cultural shift from medical paternalism toward a patient partnership model has occurred, where patients are seen as equal partners in their care [53]. Relevant studies identified that the patient-physician relationship in Iran is primarily paternalistic and physicians usually do not provide sufficient information for patients to make decisions or give them the opportunity for informed participation [54, 55]. It seems interventions are required to create a participation culture among HCPs through awareness-raising actions, co‐designing educational programs, and measuring and monitoring patient engagement activities [56].

Five main barriers at the system level were mentioned by participants, including healthcare setting-related barriers, educational barriers, lack of resources, communication barriers and organizational cultural barriers. A significant system-level barrier identified by the participants in our study was the lack of education and training for both patients and HCPs. Participants asserted the need for the inclusion of patient safety and engagement in educational curricula at the undergraduate and postgraduate levels. Previous studies emphasized that if patients are involved in patient safety, frontline HCPs need to be educated and empowered to support patient involvement [57, 58]. Common communication barriers identified by HCPs were role ambiguity,  inadequate organizational support and not being trained to engage patients. This may be eliminated by having clear policies and guidelines in place to guide HCPs on how to engage patients in safety efforts [3, 59]. Poor provider‐patient communication also perceived as another barrier to patient participation in our study, consistent with previous research [24, 60], indicating the importance of the HCPs’ ability to communicate effectively with patients and families in order to engage them in safety-related behaviors [50]. To be successful, healthcare leaders must shift the institutional culture that has historically limited patients and families' engagement to a more patient-centered culture. This requires addressing the formal policies that fail to foster patients’ involvement in safety activities as well as the informal policies that reinforce paternalistic attitudes towards patients.

Patient engagement in safety efforts is a strong priority of influential international organizations such as WHO. Over the past 20 years, interventions that encourage patients to become actively involved in their own safety have been widely implemented globally [3]. Although patient participation in the provision of safe care is an area of growing research and clinical practice in developed countries [1, 3, 13], there is limited research evidence on the acceptability of this engagement to patients and HCPs and practice evidence that such involvement leads to improvements in safety in developing countries settings. Similarly, although major efforts have been made to improve patient safety in the Iran’s hospital setting [15], there is a dearth of research on patient involvement in patient safety activities. In this context, identifying barriers and facilitators from the perspective of patients, HCPs and managers is an important first step in the selection and tailoring of interventions that could be implemented to promote and support patient particiaption. In our study, although the participants described the importance of patient involvement, many factors were recognized as hindering factors for such involvement, which were mostly similar to those reported in other contexts. This study also identified additional barriers and facilitators that may be unique to the Iran’s health system context. There is clearly a need for further applied research on how patients can best be involved and how they can act to improve the safety of care in Iran’s healthcare delivery system.

This study has several shortcomings that must be considered when interpreting the results. This study was conducted in only three teaching hospitals in Tehran and therefore, the findings cannot be generalized to other settings. Second, the scope of this study is limited to HCPs and mangers and we did not include patients. Broadening the scope could deepen our understanding of patient and family involvement processes and the challenges. Third, the focus of this study was the perceptions about patient involvement, which may be different from the actual performance of patient involvement among HCPs and managers. However, 31 individual interviews with frontline healthcare workers working in different wards, their immediate supervisors/managers and managers at the university/MoHME were conducted. Various work experience and background contributed to a large variation in the sample. This heterogeneity increased the possibility of viewing patient involvement for patient safety from different perspectives, which could be seen as a strength in the study. Inclusion of physicians in the sample would have increased the heterogeneity further.

## Conclusion

Patients and their families can play an effective role in maintaining and improving patient safety. Although there has been a significant national movement to promote patient safety in the hospital setting in Iran  during recent years, evidence is lacking on the engagement of patients in safety. A comprehensive understanding of barriers and facilitators to patient involvement in patient safety can help decision-makers and managers craft better interventions and policies to promote and support patient involvement in safety initiatives. A variety of factors were recognized to be important for involving patients and families in patient safety by managers and HCPs working in teaching hospitals in Tehran. The participants identified facilitators and barriers to patient involvement in safety-orientated activities at multiple patients, providers, and system levels, indicating that complex, multifaceted initiatives must be designed to address the issue. This study encourages further research to enhance the understating of the problems and solutions to patient involvement in safety initiatives in the Iranian healthcare setting.

## Supplementary Information

**Additional file 1.**

## Data Availability

The data sets used and analysed for the current study are available from the corresponding author on reasonable request.

## Abbreviations

HCPs: Healthcare Providers
MoHME: Ministry of Health and Medical Education
PSFHI: Patient Safety Friendly Hospital Initiative
PPE: Personal Protection Equipment
WHO: World Health Organization
