# Supplementary material for: Engaging patients in patient safety: a qualitative study examining healthcare managers and providers’ perspectives
Source: BMC Nurs. 2022 Dec 29;21:374. doi: 10.1186/s12912-022-01152-1 (PMC9801597; doi:10.1186/s12912-022-01152-1)
Supplement: Supplementary file 1 — Additional file 1. [file 12912_2022_1152_MOESM1_ESM.doc]

**Additional file 1. Topic guide of the semi-structured interviews**

**1. Respondent’s understanding of ‘patient safety’**

*Prompts*

- What do you understand by the term ‘patient safety’?

**2. Respondent 's beliefs and attitudes about patient safety**

*Prompts*

- What do you think are the risks that patients face when they go into hospital?
- Are they preventable? How much?
- What kinds of things do hospitals and staff do to keep patients safe?

**3. Thoughts about the idea that patients have a role to play in enhancing their safety during their stay at the hospital**

*Prompts*

- Your thoughts about the idea that patients have a role to play in enhancing their safety while staying in hospital?
- Do you think patients are/should be expected to help keep themselves safe when in hospital (or other health-care environment)?
- How supportive would you be of these recommended behaviours?
- Who should ultimately be responsible for patients’ safety? Why?

**4. Ways in which respondent thinks patients [or their relatives or carers] could contribute to enhancing their safety**

*Prompts*

- What sorts of things can patients do to help keep themselves safe?
- What are some examples of how patients currently participate in their hospital care?
- What needs to be done to encourage patients to do any of these things?
- What sorts of things do you think might stop patients/put them off/encourage them to do any of the things you suggest?
- Do you think relatives or carers could contribute to enhancing patient safety? How?

**5.** **Barriers and facilitators which respondent perceives to patients [or their relatives or carers] involvement in safety initiatives?**

*Prompts*

- What sorts of things can prevent patients from participating in the safety initiatives?
- What needs to be done to remove these barriers?
- What sorts of changes need at the patients’ level? healthcare providers’ level? or organizational level?
- What sorts of changes can facilitate the patients [or their relatives or carers] engagement?

**6. Anything else you would like to add?**
